# Exoscopic Extraforaminal Lumbar Interbody Fusion for Lumbar Degenerative Disease: Technical Considerations and Clinical Outcomes During the Early Learning Curve

**DOI:** 10.3390/jcm15093516

**Published:** 2026-05-04

**Authors:** Kentaro Yamane, Shinichiro Takao, Kanji Sasaki, Wataru Narita, Hisakazu Shitozawa, Kazuhiro Takeuchi, Shinnosuke Nakahara

**Affiliations:** 1 Department of Orthopedic Surgery, National Hospital Organization Okayama Medical Center, 1711-1, Tamasu, Kita-ku, Okayama 701-1192, Okayama, Japan; shinichiro.takao1225@gmail.com (S.T.); s.hisakazu0809@gmail.com (H.S.); takeuchi.kazuhiro.qr@mail.hosp.go.jp (K.T.);; 2 Department of Orthopedic Surgery, Seirei Hamamatsu General Hospital, 2-12-12, Sumiyoshi, Naka-ward, Hamamatsu 430-8558, Shizuoka, Japan; sasakan1@yahoo.co.jp; 3 Department of Orthopedic Surgery, Kameoka Municipal Hospital, 1-1, Shinonoda, Shino-cho, Kameoka 621-8585, Kyoto, Japan; wnari77@gmail.com

**Keywords:** exELIF, extraforaminal lumbar interbody fusion, exoscope, minimally invasive spinal treatment, lumbar degenerative disease

## Abstract

**Background/Objectives**: Extraforaminal lumbar interbody fusion provides indirect decompression without entering the spinal canal, but its uptake has been limited by poor visualization and risk of exiting nerve root injury. We describe a minimally invasive exoscopic extraforaminal lumbar interbody fusion (exELIF) technique and evaluate its clinical and radiological outcomes. This study aims to describe the exELIF technique and report its early clinical and radiological outcomes. **Methods**: Twenty-six patients with lumbar degenerative diseases underwent exELIF using a 3D exoscope (ORBEYE). The procedure was performed through bilateral 30–40 mm posterior incisions. Clinical outcomes were assessed using the Japanese Orthopedic Association score preoperatively and at 1-year follow-up. Postoperative computed tomography evaluated interbody fusion. Operative time, blood loss, and complications were recorded. **Results**: Mean operative time was 131 ± 51 min, and mean estimated blood loss was 82 ± 99 mL. The mean JOA score improved from 15.2 ± 2.2 to 24.3 ± 2.6, with a mean recovery rate of 66% at 1 year. Interbody fusion was achieved in 96%. In an exploratory CUSUM analysis of 18 single-level fluoroscopy-guided cases, a transition in operative time was observed at approximately the 10th case; operative time and estimated blood loss decreased from 141.5 ± 39.2 min and 89.0 ± 77.8 mL in cases 1–10 to 80.1 ± 6.7 min and 21.2 ± 18.1 mL in cases 11–18 (*p* < 0.001 and *p* = 0.035, respectively), indicating a reduction of operative time with accumulated experience rather than a formally established learning curve. Three patients developed transient exiting nerve root symptoms that resolved spontaneously during follow-up. One patient at the L5/S level required revision surgery due to left L5 nerve root palsy caused by posterior migration of the bone graft; this complication led to a modification of the technique, with posterior bone grafting no longer performed at L5/S. Partial screw loosening was observed in 5 patients (19%), all of which were asymptomatic and required no additional intervention. **Conclusions**: ExELIF provides excellent visualization in deep surgical fields, allowing the use of conventional surgical instruments through minimally invasive incisions. This is an early feasibility report of a single-institution retrospective case series with a heterogeneous cohort and no control group; the present data therefore do not establish superiority over conventional or endoscopic ELIF. Within these limits, exELIF was associated with acceptable early clinical improvement and a high interbody fusion rate, and progressive reduction in operative time with experience suggests that it may be a technically feasible minimally invasive option for selected patients with lumbar degenerative disease and for revision surgery after lumbar decompression.

## 1. Introduction

Low back pain and lumbar degenerative disorders are among the most common causes of functional impairment and reduced quality of life, representing a major public health burden worldwide [1]. Lumbar interbody fusion is a surgical procedure performed to treat degenerative lumbar diseases that are refractory to conservative management. Various approaches, including anterior lumbar interbody fusion, posterior lumbar interbody fusion (PLIF), transforaminal lumbar interbody fusion (TLIF), and lateral lumbar interbody fusion (LLIF), have been utilized. Advances in surgical instruments have helped minimize the invasiveness of each technique.

ELIF was first reported by Phillip et al. in 2002 [2]. This approach, which avoids direct access to the spinal canal, is considered effective not only for degenerative lumbar diseases but also for revision surgery after decompression. However, because of the depth of the surgical field, visualization is challenging when performed through a small incision, and the risk of exiting nerve root injury has limited its wider adoption [3]. In recent years, advances in endoscopic technology have enhanced visualization, prompting an increasing number of reports on endoscopic-assisted ELIF [4,5,6]. However, these procedures involve a steep learning curve, require dedicated endoscopic instruments, and may cause surgeons to lose anatomical orientation due to the restricted two-dimensional endoscopic view. Consequently, a technique that combines the minimal invasiveness of endoscopic approaches with the familiar orientation and instrumentation of open surgery has remained an unmet clinical need.

Exoscopes are widely used as alternative magnification devices to microscopes. Equipped with a compact, highly maneuverable camera head, an exoscope provides a broader working space [7,8] while allowing surgeons to view high-resolution 3D images on a 4K monitor using 3D polarized glasses. We previously reported exoscopic minimally invasive open-door laminoplasty performed through a 30-mm skin incision [9]. The use of an exoscope enables excellent visualization, even in deep surgical fields, with minimal incisions, making it a viable option for the ELIF approach. Unlike endoscopic systems, which require specialized instruments and a steep learning curve, the exoscope allows the use of conventional open surgical tools, potentially lowering the barrier to adoption. Building on our previous experience with exoscopic spinal surgery [9], this study aimed to introduce a novel minimally invasive exoscopic ELIF (exELIF) technique for the treatment of lumbar degenerative diseases and to evaluate its clinical outcomes and learning curve. The true novelty of exELIF lies in combining skin incisions comparable to endoscopic ELIF with a mini-open working corridor that preserves three-dimensional anatomical orientation, enables the use of conventional open surgical instruments rather than dedicated endoscopic tools, and is expected to shorten the learning curve compared with endoscopic techniques.

## 2. Materials and Methods

This study included 26 patients who underwent exELIF for degenerative lumbar pathology using the ORBEYE exoscope (Sony Olympus Medical Solutions, Tokyo, Japan). The study was approved by the Institutional Review Board of our institute, and written informed consent was obtained from all participants.

### 2.1. Surgical Indication

The indications for exELIF included lumbar degenerative diseases such as degenerative spondylolisthesis, foraminal stenosis, low-grade isthmic spondylolisthesis, revision surgery following lumbar decompression, and degenerative scoliosis at the L1–S1 level.

### 2.2. Contraindications

Patients requiring direct decompression or dural manipulation owing to neurological deficits or ossification of the posterior longitudinal ligament or ligamentum flavum.

### 2.3. Description of the Surgical Technique

#### 2.3.1. Approach to Kambin’s Safety Triangle

**Figure 1:**
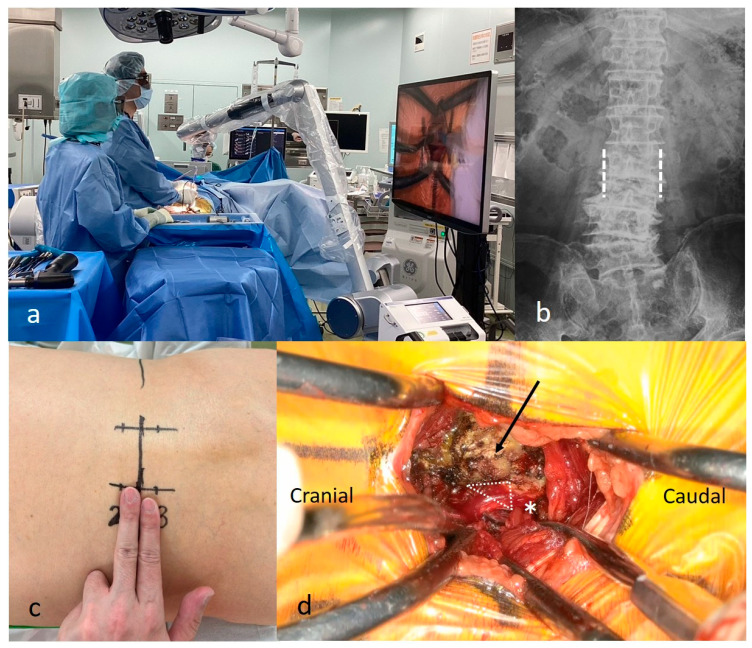
Surgical setting and initial approach during exoscopic extraforaminal lumbar interbody fusion. (**a**) The camera is placed above the surgical field, and the surgeon, wearing three-dimensional polarized glasses, performs the procedure while viewing the monitor. (**b**) Bilateral 30–40 mm incisions are made at the outer edge of the vertebra, centered at the disc level. The dashed line indicates the skin incision site. (**c**) The procedure is performed through two small incisions, each approximately two fingerbreadths in length. (**d**) The facet joint (black arrow) is exposed, and the caudal transverse process (*) is palpated with a mucosal elevator. Kambinʼs safety triangle is identified (dotted triangle).

Patients were placed in the prone position on a radiolucent carbon table. An exoscopic camera was positioned above the surgical field, and the surgeon wore 3D polarized glasses to perform the procedure while viewing 3D images on a 4K large-screen monitor (Figure 1a). Bilateral incisions of 30–40 mm were made at the outer edge of the vertebra, centered at the disc level (Figure 1b,c). After incising the fascia, the facet joint between the lumbar multifidus and longissimus muscles was exposed by blunt dissection using a finger and an electric scalpel. Two deep gelpi retractors were used to retract the muscles, allowing clear visualization. The caudal transverse process was palpated with a mucosal elevator, and the Kambinʼs safety triangle was identified (Figure 1d).

#### 2.3.2. Partial Resection of the Facet Joint and Discectomy (Figure 2a,b)

**Figure 2:**
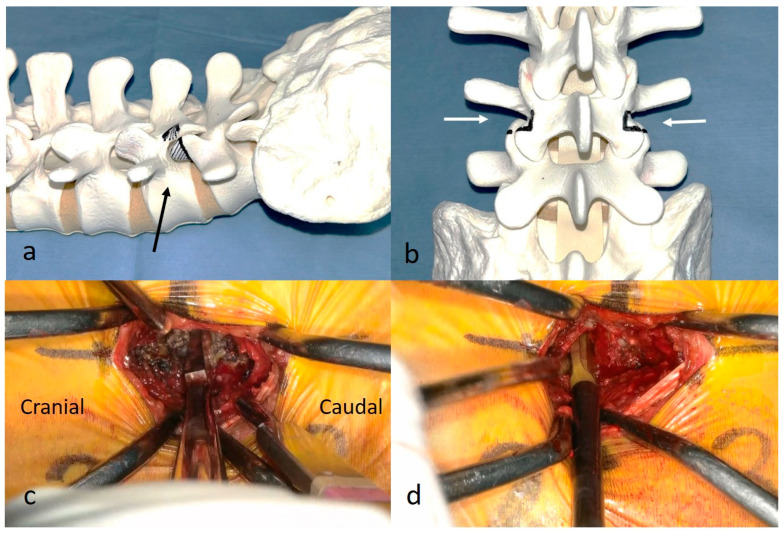
Partial resection of the facet joint and discectomy. (**a**,**b**) Resection lines of the superior and inferior articular processes in the bone model (black arrow). The entire superior articular process and the lateral portions of the inferior articular processes were resected bilaterally (white arrows). (**c**) Resection of the superior articular process using a chisel. (**d**) Discectomy is performed using disc shavers and curettes.

After resecting the entire superior articular process (SAP) using a high-speed burr and chisel (Figure 2c), the lateral portion of the inferior articular process (IAP) was also resected using a Kerrison rongeur as needed. The resected SAP and IAP were milled and used as graft bones. The exiting nerve root was not routinely exposed. Discectomy was performed using disc shavers and both straight and curved curettes (Figure 2d), aiming for thorough removal of disc material.

#### 2.3.3. Bone Grafting and Cage Insertion

**Figure 3:**
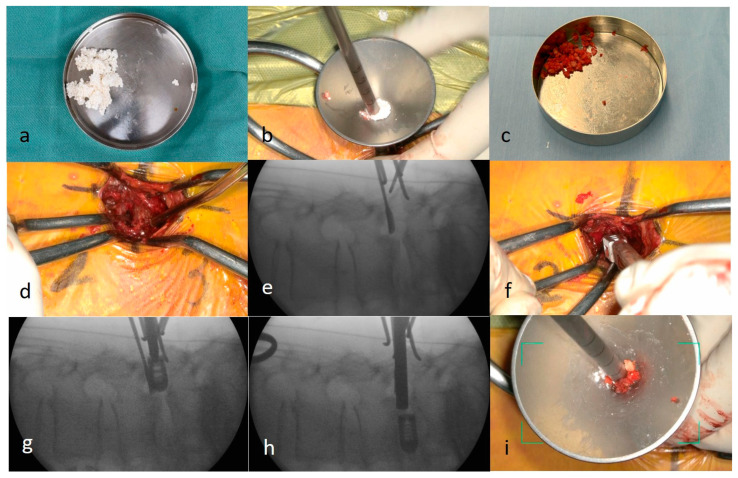
Bone grafting and cage insertion. (**a**) Preparation of an artificial bone graft. (**b**) Placement of an artificial bone graft into the intervertebral space. (**c**) Milled local bone graft harvested from the superior articular process and the lateral portion of the inferior articular process. (**d**) The exiting nerve root is indirectly protected using a nerve root retractor. (**e**) Proper positioning of the nerve root retractor verified using C-arm fluoroscopy. (**f**) Insertion of the cage packed with milled local bone graft. (**g**) Under fluoroscopic guidance, the nerve root retractor protects the exiting nerve root during cage insertion. (**h**,**i**) The remaining local bone is grafted posterior to the cage.

Artificial bone grafts (NovoGro, Sydney, Australia; OsteoNovus, Inc., Toledo, OH, USA), along with milled local bone grafts, were placed in the intervertebral space (Figure 3a–c). During cage insertion, the exiting nerve and surrounding soft tissues were indirectly protected using a nerve root retractor (Figure 3d,e). A cage packed with a milled local bone graft was inserted under C-arm fluoroscopic guidance (Figure 3f,g). The remaining local bone was grafted behind the cage (Figure 3h,i). The same procedure was performed on the contralateral side.

#### 2.3.4. Percutaneous Pedicle Screw Procedure

**Figure 4:**
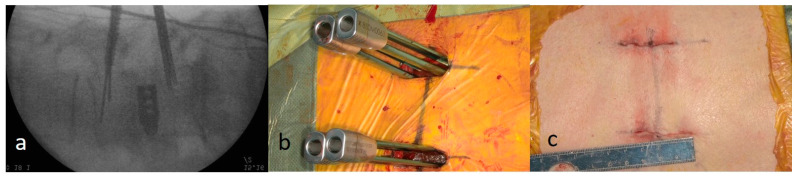
Percutaneous pedicle screw procedure under fluoroscopic guidance. (**a**,**b**) Screw insertion performed under fluoroscopic guidance using the percutaneous pedicle screw technique. (**c**) The bilateral incisions are closed without placement of a drainage tube.

Fluoroscopic or intraoperative computed tomography (CT) navigation was used for guidance. In this series, percutaneous pedicle screws (PPSs) were inserted under fluoroscopic guidance in 21 cases and with the aid of intraoperative CT navigation in five cases. Under fluoroscopic guidance, needles were bilaterally inserted through the original lateral incisions (Figure 4a). After guidewire insertion, a cannulated tap was used to create a screw pathway. Cannulated pedicle screws were inserted over the guide wires into the vertebrae (Figure 4b). When intraoperative CT navigation was used, the reference pins for the navigation system were inserted into the spinous process and secured in place. After acquiring the navigation scan, probing and tapping were performed under navigational guidance, followed by screw insertion. Subsequently, rods were guided through the screw tabs and secured with screws. In cases of degenerative lumbar spondylolisthesis, vertebral slippage was effectively corrected using screw devices. The two incisions were irrigated, and each tissue layer was closed without placing a drainage tube (Figure 4c).

A surgical short video of ExELIF is available online as Supplementary Materials.

Operating time and estimated blood loss were also recorded. The Japanese Orthopedic Association (JOA) score for low back pain was assessed preoperatively and at the 1-year follow-up. The recovery rate was calculated using the Hirabayashi formula: (postoperative JOA score)/(29 − preoperative JOA score) × 100%. Postoperative CT scans were obtained for all patients, and bone fusion was evaluated using CT images acquired 1 year after surgery. Fusion status was assessed at 1 year on coronal and sagittal CT reconstructions and classified according to the criteria of Tan et al. [10], in which Grade I represents complete bridging trabecular bone across the disc space, Grade II partial bridging trabecular bone, Grade III unilateral bridging without complete fusion, and Grade IV no bridging or pseudarthrosis; Grades I and II were considered solid fusion. Two of the authors (the operating surgeon and an independent reviewer not involved in the surgery) assessed the images independently, and disagreements were resolved by consensus. Surgery-related complications were also evaluated. To evaluate the learning curve of the exELIF procedure under standardized conditions, a subgroup analysis was performed. Only patients who underwent single-level fusion with percutaneous pedicle screw insertion under fluoroscopic guidance were included to minimize the influence of case complexity and navigation use. A total of 18 cases met these criteria. These cases were arranged in chronological order. To objectively determine the transition from the learning phase to the proficiency phase, a cumulative sum (CUSUM) analysis of operative time was performed. The mean operative time of the 18 cases was used as the target value, and the cumulative sum of deviations from the target value was plotted against the case number. The case number corresponding to the peak of the CUSUM curve was defined as the inflection point separating the early (learning) phase from the late (proficiency) phase. Operative time and estimated blood loss were compared between the two groups. Continuous variables are presented as mean ± standard deviation and were compared using the Mann–Whitney U test. A *p*-value < 0.05 was considered statistically significant. All statistical analyses were performed using StatMate version 5 (ATMS Co., Ltd., Tokyo, Japan).

## 3. Results

Table 1 summarizes the clinical information of the 26 patients who underwent exELIF. Of these, 22 patients underwent single-level exELIF, two underwent two-level exELIF, and two underwent single-level exELIF combined with microendoscopic laminectomy (MEL) at the adjacent level. The mean operating time was 131 ± 51 min (range, 65–261 min), and the mean intraoperative blood loss was 82 ± 99 mL (range, 10–420 mL). The mean JOA score improved from 15.2 ± 2.2 to 24.3 ± 2.6, with a mean recovery rate of 66%. The intervertebral bone fusion rate was 96%. Interbody bone fusion was observed within the cage in 23 patients (88%), posterior to the cage in 20 patients (77%), and lateral to the intervertebral space in 6 patients (23%); these categories were not mutually exclusive. Partial screw loosening occurred in 5 patients (19%); all affected patients were asymptomatic and did not require additional surgical intervention. Exiting nerve root disorders, such as lower extremity pain, developed in three patients during cage insertion but improved spontaneously during follow-up. In one patient at the L5/S level, revision surgery was performed to remove the grafted bone owing to left L5 nerve root palsy caused by the posterior migration of the bone graft behind the cage; as a result, posterior bone grafting behind the cage at the L5/S level was subsequently abandoned to avoid similar complications.

**Figure 5:**
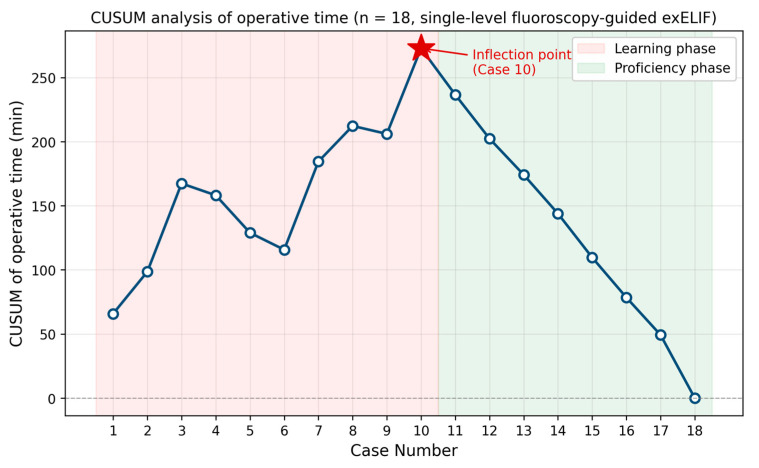
CUSUM (cumulative sum) analysis of operative time for the 18 single-level fluoroscopy-guided exELIF cases. The CUSUM curve peaks at the 10th case (red star), which was defined as the inflection point separating the learning phase (cases 1–10; pink shading) from the proficiency phase (cases 11–18; green shading).

To evaluate the learning curve under standardized conditions, only single-level cases with percutaneous pedicle screw insertion performed under fluoroscopic guidance were analyzed (*n* = 18) (Table 2). CUSUM analysis of operative time identified an inflection point at the 10th case (Figure 5). Based on this inflection point, the 18 cases were divided into an early (learning) phase (cases 1–10) and a late (proficiency) phase (cases 11–18). The mean operative time decreased significantly from 141.5 ± 39.2 min in the early phase to 80.1 ± 6.7 min in the late phase (*p* < 0.001). The mean estimated blood loss also decreased significantly from 89.0 ± 77.8 mL in the early phase to 21.2 ± 18.1 mL in the late phase (*p* = 0.035). These findings indicate that surgical efficiency, in terms of both operative time and estimated blood loss, improved markedly after approximately 10 cases.

**Figure 6:**
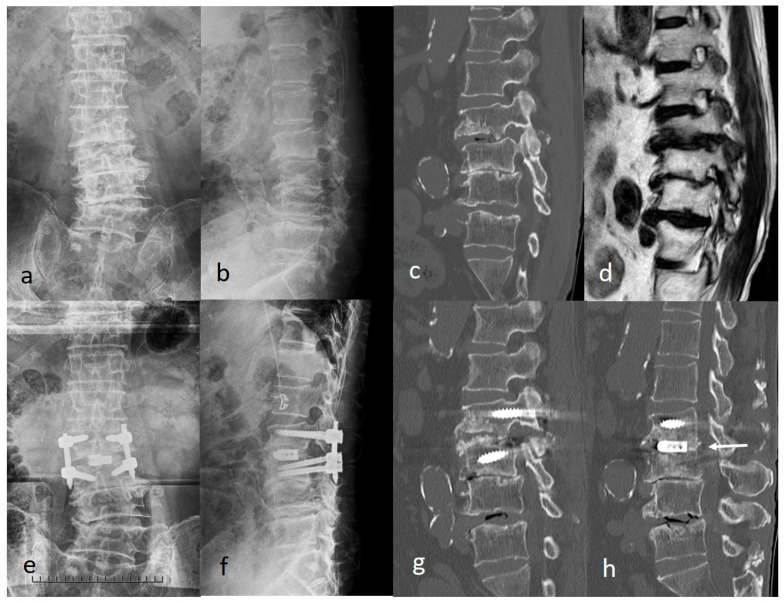
Case of left-sided foraminal stenosis at the L2/3. (**a**,**b**) Preoperative radiograph. (**c**,**d**) Preoperative computed tomography and magnetic resonance imaging revealing left-sided foraminal stenosis at L2/3. (**e**,**f**) Postoperative radiograph. (**g**) Postoperative computed tomography revealed a significant reduction in foraminal stenosis. (**h**) Intervertebral bone fusion posterior to the cage (white arrow).

Case 1: A 77-year-old man presented with left leg and lower back pain. Radiographs revealed degenerative changes in the lumbar spine (Figure 6a,b). CT and magnetic resonance imaging (MRI) revealed left-sided foraminal stenosis at the L2/3 level (Figure 6c,d). ExELIF was performed through 35-mm bilateral incisions at the outer edge of the vertebra, centered at the L2/3 disc level (Figure 6e,f). Postoperative CT revealed a significant reduction in foraminal stenosis (Figure 6g). No postoperative complications were observed, and the JOA score improved from 13 to 25 1 year postoperatively. Intervertebral bone fusion was observed posterior to the cage 1 year postoperatively (Figure 6h).

**Figure 7:**
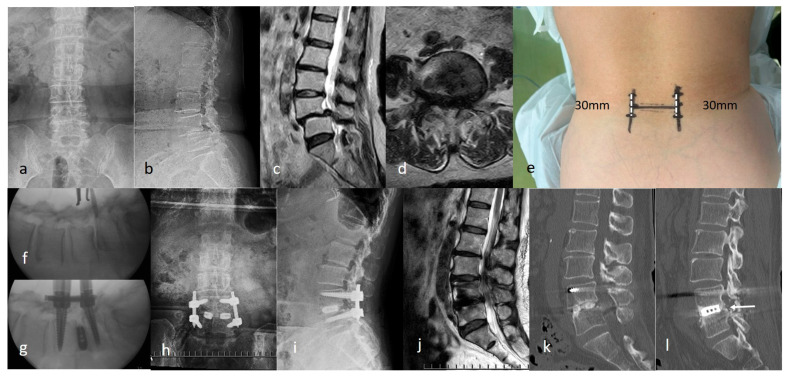
Case of grade 2 spondylolisthesis at L4/5. (**a**,**b**) Preoperative radiograph. (**c**,**d**) Preoperative magnetic resonance images revealing spinal canal stenosis at L4/5. (**e**) The procedure was performed through 30-mm bilateral incisions. (**f**,**g**) Reduction of the slippage using pedicle screw devices. (**h**,**i**) Postoperative radiograph. (**j**,**k**) Postoperative magnetic resonance and computed tomography images showing a significant reduction in slippage and canal stenosis. (**l**) Intervertebral bone fusion posterior to the cage (white arrow).

Case 2: A 77-year-old woman presented with bilateral sciatica and intermittent claudication. Radiographs revealed grade 2 spondylolisthesis at the L4/5 (Figure 7a,b), and an MRI showed spinal canal stenosis at the same level (Figure 7c,d). ExELIF was performed through 30-mm bilateral incisions at the outer edge of the vertebra, centered at the L4/5 disc level (Figure 7e). Slippage was effectively corrected using screw devices (Figure 7f,g). Postoperative radiographs, CT, and MRI revealed a significant reduction in slippage and canal stenosis (Figure 7h–k). No postoperative complications were observed, and the JOA score improved from 19 to 29 1 year postoperatively. Intervertebral bone fusion posterior to the cage was achieved at 1 year postoperatively (Figure 7l).

**Figure 8:**
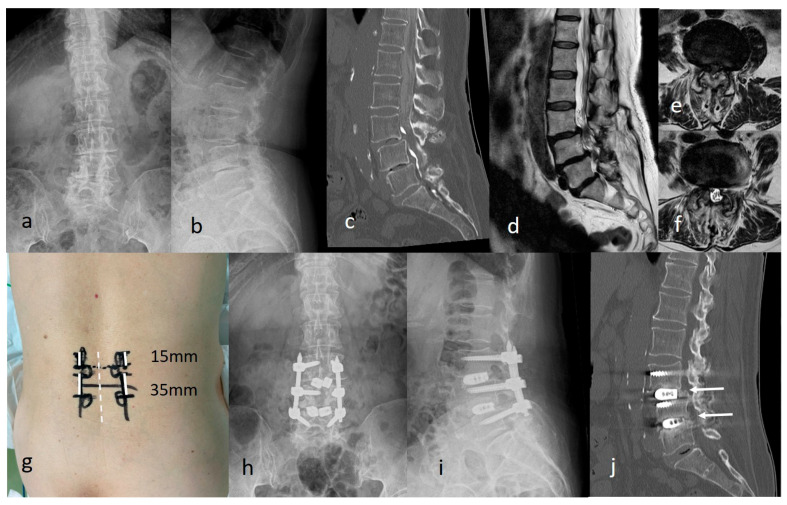
Revision case after lumbar decompression at L3/4 and L4/5. (**a**,**b**) Preoperative radiograph. (**c**–**f**) Preoperative computed tomography and magnetic resonance images showing spinal canal stenosis at L3/4 and L4/5. (**g**) The procedure is performed through four bilateral incisions (white solid line) without entering the spinal canal at the site of the previous surgery (white dotted line). (**h**,**i**) Postoperative radiograph. (**j**) Intervertebral bone fusion was achieved posterior to the cage at both levels (white arrows).

Case 3: A 75-year-old woman underwent partial laminectomy with medial facetectomy at L3–L5 for lumbar spinal canal stenosis 14 years before presentation. Subsequently, she developed lower back and bilateral leg pain and visited our department. Radiographs revealed grade 1 spondylolisthesis at L3 and L4 (Figure 8a,b). CT myelography and MRI confirmed spinal canal stenosis at the same level (Figure 8c–f). Two-level exELIF was performed through four bilateral incisions without entering the spinal canal at the site of the previous surgery (Figure 8g). L3 pedicle screws were inserted through the cranial incisions, whereas L4 and L5 screws, along with interbody procedures at L3/4 and L4/5, were inserted through the caudal incisions. Vertebral slippage was corrected using a screw system. Postoperative radiographs, CT, and MRI demonstrated a significant reduction in slippage and canal stenosis (Figure 8h,i). No postoperative complications were observed. The JOA score improved from 10 to 26 at 1 year postoperatively. Interbody bone fusion at both levels was achieved 1 year postoperatively (Figure 8j).

## 4. Discussion

ExELIF is a surgical procedure designed to improve symptoms via indirect decompression. Indirect decompression of the lumbar spine does not require manipulation within the spinal canal, offering advantages such as reduced intraoperative blood loss and avoidance of postoperative epidural hematoma [11]. The efficacy of indirect decompression has been demonstrated in lumbar fusion surgery using LLIF techniques [12]. Compared with conventional PLIF, LLIF is less invasive yet achieves equivalent clinical outcomes [13]. In addition, it shortens hospitalization duration and reduces perioperative complications [14]. ELIF is another approach that accesses the intervertebral space from the foraminal side, avoiding direct exposure of the spinal canal and achieving indirect decompression. Unlike LLIF, ELIF does not require a lateral abdominal incision and can be performed solely through posterior skin incisions, making it less invasive. In addition, ELIF eliminates the need for patient repositioning required in standard LLIF procedures. Various ELIF techniques utilizing endoscopy and microendoscopy, such as PETLIF (percutaneous endoscopic transforaminal lumbar interbody fusion) [4], UBE-LIF (unilateral biportal endoscopy-assisted extraforaminal lumbar interbody fusion) [5], and microendoscopy-assisted extraforaminal lumbar interbody fusion [6], have been reported. ExELIF, which utilizes an exoscope, provides a wider working space than endoscopic ELIF. This technique allows the use of surgical instruments and procedures similar to those used in conventional open surgery, which may shorten the time required for surgeons to gain procedural familiarity. When the analysis was limited to single-level cases performed under fluoroscopic guidance (*n* = 18), exploratory CUSUM analysis identified a transition point in operative time at approximately the 10th case. Operative time and estimated blood loss decreased from 141.5 ± 39.2 min and 89.0 ± 77.8 mL in cases 1–10 to 80.1 ± 6.7 min and 21.2 ± 18.1 mL in cases 11–18 (*p* < 0.001 and *p* = 0.035, respectively). These findings should be interpreted as a reduction of operative time with accumulated surgical experience, rather than as a formally established learning curve, given the small sample size and the absence of a comparator cohort. This relatively rapid reduction in operative time may be attributed to the use of conventional surgical instruments and a familiar operative view compared with endoscopic techniques; however, because the present analysis was based on only 18 cases, it should not be interpreted as a formally established learning curve. Furthermore, exELIF can be performed through skin incisions of approximately 30–40 mm on each side, which is comparable to the PPS technique.

In this study, exELIF was primarily performed for degenerative lumbar spondylolisthesis, foraminal stenosis, and revision surgery after lumbar decompression. Most cases involved single-level procedures; however, exELIF is also feasible for multilevel surgery. Nevertheless, for deformity correction across three or more spinal levels, LLIF may be more advantageous, as it allows simultaneous multilevel cage insertion through retroperitoneal exposure and enables the use of larger cages, providing stronger corrective force. For revision surgery after lumbar decompression, neither exELIF nor LLIF requires manipulation within the spinal canal, allowing a safer approach without disrupting adhesions. In cases of foraminal stenosis and wedge deformities, craniocaudal narrowing of the foramen increases the risk of injury to the exiting nerve root during the extraforaminal approach. However, exELIF enables safe cage insertion by securely protecting the exiting nerve root using a commercially available nerve root retractor. In contrast, during endoscopic ELIF, the exiting nerve root cannot be directly protected using a conventional nerve root retractor, necessitating specialized techniques, such as using specialized sliders or expandable cages to prevent nerve injury during cage insertion [15].

In ExELIF, the SAP is completely resected, whereas extensive resection of the lateral portion of the IAP allows for a more medial cage insertion. This creates a safer zone farther from the exiting nerve root while preserving the medial portion of the IAP, thereby preventing contact with the dura [16]. Endoscopic ELIF, such as PETLIF, faces challenges at the L5/S level due to interference between the endoscope and the iliac crest. However, exELIF using an exoscope can be performed at the L5/S level using the same approach as at other intervertebral levels.

With advancements in surgical techniques and implant technology, lumbar interbody fusion procedures now routinely achieve fusion rates > 90% [17,18]. One challenge of ELIF is that, compared with conventional PLIF, it yields less local bone for grafting, necessitating modifications to the bone grafting technique. Both the entire SAP and lateral portions of the IAP were resected in all cases to maximize the local bone harvest. Previous studies reported fusion rates of 88% for PETLIF and 78% for UBE-LIF [4,5], which are lower than those achieved with conventional LIF procedures. In this study, a bone fusion rate of 96% was achieved by grafting artificial bone into the intervertebral space and transplanting a limited amount of local bone inside and behind the cage without the need for additional iliac bone harvesting. Xu et al. reported the advantage of achieving fusion in the posterior part of the cage, attributing it to appropriate preparation of the posterior endplate and the greater biomechanical stability of the posterior zone [19]. In ExELIF, bone grafting is performed in the posterior area of the cage, increasing the likelihood of fusion in the posterior zone. In this study, bone union posterior to the cage was observed in 77% of cases. However, at the L5/S level, one patient developed L5 nerve root palsy due to posterior migration of the bone graft behind the cage, necessitating revision surgery. This complication was attributed to the anatomical characteristics of the L5/S level. Compared to other levels, the vertical diameter of the intervertebral foramen at L5/S was relatively smaller, and the mobility of the L5 nerve root was limited owing to the influence of the sacral ala and L5 transverse process. Consequently, posterior bone grafting behind the cage at the L5/S level was not performed to avoid similar complications. In the index case, local bone graft was intentionally placed posterior to the cage to enhance fusion; however, because of the narrow L5/S foramen and the restricted mobility of the L5 nerve root caused by the sacral ala and L5 transverse process, even minor posterior migration of the grafted fragments is sufficient to compress the exiting nerve root. This case therefore taught us that, at the L5/S level, any graft placed behind the cage carries a high risk of migrating into the foramen and producing nerve root injury. Based on this experience, we now refrain from posterior bone grafting at L5/S, restricting graft placement to the inside and anterior aspects of the cage. Consistent use of a nerve root retractor during cage insertion, together with avoidance of overpacking the posterior portion of the disc space, is also considered essential to prevent similar complications.

The use of intraoperative fluoroscopy, including the PPS technique, increases radiation exposure to the surgeon. Implementation of intraoperative CT navigation can help reduce radiation exposure [20]. The overall complication profile of exELIF in the present cohort—transient exiting nerve root symptoms in 3 of 26 patients (12%), one L5 nerve root palsy requiring revision (4%), and asymptomatic partial screw loosening in 5 patients (19%)—is broadly comparable with previously reported rates for other minimally invasive fusion techniques. PETLIF has reported transient exiting nerve root symptoms in approximately 8% of cases [4], and pedicle screw loosening, particularly in older patients with reduced bone quality, has been described at variable rates in the literature, generally ranging from approximately 1% in younger patients to more than 15% in osteoporotic populations [21]. Importantly, no dural tears, wound infections, or epidural hematomas were encountered in our series, which may reflect the avoidance of intracanalicular manipulation intrinsic to the extraforaminal approach. Nonetheless, because exELIF accesses the intervertebral space through Kambin’s triangle, exiting nerve root irritation remains the most relevant safety concern and should be the principal focus of complication surveillance as this technique is adopted more widely.

This study had several limitations. First, it was a single-institution, retrospective case series without a control group, and no direct comparison was made with established techniques such as TLIF, LLIF, or endoscopic ELIF; consequently, the superiority or equivalence of exELIF cannot be established from the present data. Second, the sample size was small (*n* = 26) and the indications were heterogeneous (degenerative and isthmic spondylolisthesis, foraminal stenosis, and revision surgery), which limits the statistical power of subgroup analyses and the generalizability of the findings. Third, the learning curve analysis was based on 18 single-level fluoroscopy-guided cases; although the CUSUM-based inflection point provides a more robust estimate than an arbitrary even split, the absolute number of cases remains limited and the curve described here should be regarded as preliminary. Fourth, clinical outcomes were assessed using the JOA score alone, and validated patient-reported outcome measures such as the Visual Analogue Scale, Oswestry Disability Index, and health-related quality-of-life instruments were not collected; inclusion of these measures in future studies would allow a more comprehensive assessment of clinical benefit. Fifth, the follow-up period was limited to 1 year, which is short for the evaluation of fusion durability, adjacent segment disease, and late screw-related complications; longer-term follow-up is therefore essential. Accordingly, prospective, multicenter comparative studies with larger cohorts, standardized indications, multiple validated outcome measures, and extended follow-up are warranted to confirm the efficacy, safety, and generalizability of exELIF. Within these constraints, the present findings suggest that exELIF is a feasible and potentially useful minimally invasive option for selected patients with lumbar degenerative disease.

## 5. Conclusions

By using an exoscope, exELIF can be performed through minimally invasive skin incisions comparable to those used in the PPS technique, allowing its application at all lumbar levels, including L5/S. In an exploratory CUSUM analysis, a transition in operative time was observed at approximately the 10th case, and both operative time and estimated blood loss decreased significantly thereafter; these findings should be interpreted as a reduction of operative time with accumulated experience rather than as a formally established learning curve. Within the limitations of this retrospective, single-center case series, exELIF appears to be a feasible minimally invasive interbody fusion technique with acceptable early clinical and radiological outcomes for selected patients with lumbar degenerative disease and for revision surgery after lumbar decompression. These preliminary findings should be interpreted with caution, and prospective comparative studies with larger cohorts and longer follow-up are warranted to confirm the clinical value and generalizability of this technique.

## Figures and Tables

**Table 1:** Characteristics and surgical details of patients who underwent exoscopic extraforaminal lumbar interbody fusion.

No.	Age (yrs)	Sex	Diagnosis	Fusion Level	PPS Insertion	Operating Time (min)	Blood Loss (mL)	JOA Score		Bone Fusion	Bone Fusion Site			Complications/ Screw Loosening
								Pre	1 Year		L	P	W	
1	81	F	DS	L4/5	Flu	180	160	17	28	+		+	+	
2	85	M	DS	L4/5	Flu	147	150	16	26	+	+	+	+	ENR
3	66	F	DS	L4/5	Flu	183	60	16	24	+	+	+	+	
4	48	F	DS	L4/5	Flu	105	40	15	22	+			+	
5	77	M	FS	L2/3	Flu	85	10	13	25	+		+	+	
6	62	F	DS	L4/5	Flu	101	220	17	27	+		+	+	
7	36	F	DS	L5/S	Flu	183	20	16	27	+		+	+	
8	77	F	DS	L4/5	Flu	142	170	19	29	+		+	+	
9	82	F	FS	L5/S	Flu	108	10	16	26	+	+	+	+	1 screw loosening
10	55	M	DS	L4/5	Flu	181	50	17	26	+		+	+	
11	77	F	DS	L4/5	Flu	78	10	14	23	+		+	+	1 screw loosening
12	75	F	RS	L3/4/5	Flu	189	265	10	26	+		+	+	
13	61	F	DS	L4/5	Navi	161	50	14	25	+	+			
14	86	F	DS	L4/5	Navi	191	90	13	24	+	+			ENR
15	75	F	DS	L4/5	Flu	80	50	17	25	+	+	+	+	
16	77	M	FS	L4/5	Flu	86	10	12	22	+		+	+	
17	79	F	FS	L5/S	Flu	84	20	16	19	+		+	+	2 screws loosening
18	74	M	FS	L5/S	Flu	80	50	16	22	+		+	+	2 screws loosening
19	79	M	DS	L4/5	Flu	83	10	17	24	+		+	+	
20	73	M	L5/S FS L4/5 LCS	L5/S L4/5 MEL	Flu	132	10	13	25	+		+	+	
21	52	M	FS	L4/5	Flu	85	10	15	26	+		+	+	
22	82	F	DS	L2/3	Flu	65	10	18	24	+		+	+	
23	68	F	FS	L5/S	Navi	113	40	17	19	+			+	* Revision surgery
24	84	F	RS	L3/4/5	Navi	261	420	13	20					2 screws loosening
25	72	M	L5/S FS L3/4 LCS	L5/S L3/4 MEL	Flu	191	90	17	22	+			+	ENR
26	63	F	DS	L4/5	Navi	113	100	11	27	+		+	+	

DS: Degenerative spondylolisthesis, FS: Foraminal stenosis, RS: Revision surgery after lumbar decompression, LCS: Lumbar canal stenosis. MEL: Microendoscopic laminectomy. Flu: Fluoroscopic, Navi: intraoperative CT navigation. L: Lateral portion, P: Posterior to the cage, W: Within the cage. ENR: Exiting nerve root disorder. * Revision surgery was performed because of nerve root injury caused by the posterior migration of the bone graft.

**Table 2:** Comparison of operative time and estimated blood loss between the early (learning) phase (cases 1–10) and the late (proficiency) phase (cases 11–18), divided according to the CUSUM inflection point, in single-level exELIF performed under fluoroscopic guidance.

	Early Phase (Cases 1–10)	Late Phase (Cases 11–18)	*p*-Value
Operating time (min)	141.5 ± 39.2	80.1 ± 6.7	<0.001
Intraoperative blood loss (mL)	89.0 ± 77.8	21.2 ± 18.1	0.035

## Data Availability

The data used to support the findings of this study are available from the corresponding authors upon request.

## Supplementary Materials

The following supporting information can be downloaded at: https://www.mdpi.com/article/10.3390/jcm15093516/s1, Video S1: Surgical short video.

## Abbreviations

The following abbreviations are used in this manuscript:

ELIF	Extraforaminal lumbar interbody fusion
TLIF	Transforaminal lumbar interbody fusion
PLIF	Posterior lumbar interbody fusion
LLIF	Lateral lumbar interbody fusion
exELIF	Exoscopic extraforaminal lumbar interbody fusion
PPS	Percutaneous pedicle screw
CT	Computed tomography
SAP	Superior articular process
IAP	Inferior articular process
JOA	The Japanese Orthopedic Association
MEL	Microendoscopic laminectomy
MRI	Magnetic resonance imaging
PETLIF	Percutaneous endoscopic transforaminal lumbar interbody fusion
